# How executives’ expectations and experiences shape population health management strategies

**DOI:** 10.1186/s12913-019-4513-3

**Published:** 2019-10-26

**Authors:** Betty M. Steenkamer, Hanneke W. Drewes, Natascha van Vooren, Caroline A. Baan, Hans van Oers, Kim Putters

**Affiliations:** 1 Tilburg School of Social and Behavioural Sciences, Tilburg University, Tranzo, PO Box 90153, 5000 LE Tilburg, The Netherlands; 2 National Institute for Public Health and the Environment (RIVM), PO Box 1, 3720 BA Bilthoven, The Netherlands; 3 Erasmus School of Health Policy & Management (ESHPM), P.O. Box 1738, 3000 DR Rotterdam, The Netherlands; 4 The Netherlands Institute for Social Research, PO Box 16164, 2500 BD The Hague, The Netherlands

**Keywords:** Population health management strategies, Realist method, Executives’ expectations

## Abstract

**Background:**

Within Population Health Management (PHM) initiatives, stakeholders from various sectors apply PHM strategies, via which services are reorganised and integrated in order to improve population health and quality of care while reducing cost growth. This study unravelled how stakeholders’ expectations and prior experiences influenced stakeholders intended PHM strategies.

**Methods:**

This study used realist principles. Nine Dutch PHM initiatives participated. Seventy stakeholders (mainly executive level) from seven different stakeholder groups (healthcare insurers, hospitals, primary care groups, municipalities, patient representative organisations, regional businesses and program managers of the PHM initiatives) were interviewed. Associations between expectations, prior experiences and intended strategies of the various stakeholder groups were identified through analyses of the interviews.

**Results:**

Stakeholders’ expectations, their underlying explanations and intended strategies could be categorized into four themes: 1. Regional collaboration; 2. Governance structures and stakeholder roles; 3. Regional learning environments, and 4. Financial and regulative conditions. Stakeholders agreed on the long-term expectations of PHM development. Differences in short- and middle-term expectations, and prior experiences were identified between stakeholder groups and within the stakeholder group healthcare insurers. These differences influenced stakeholders’ intended strategies. For instance, healthcare insurers that intended to stay close to the business of care had encountered barriers in pushing PHM e.g. lack of data insight, and expected that staying in control of the purchasing process was the best way to achieve value for money. Healthcare insurers that were more keen to invest in experiments with data-technology, new forms of payment and accountability had encountered positive experiences in establishing regional responsibility and expected this to be a strong driver for establishing improvements in regional health and a vital and economic competitive region.

**Conclusion:**

This is the first study that revealed insight into the differences and similarities between stakeholder groups’ expectations, experiences and intended strategies. These insights can be used to improve the pivotal cooperation within and between stakeholder groups for PHM.

**Electronic supplementary material:**

The online version of this article (10.1186/s12913-019-4513-3) contains supplementary material, which is available to authorized users.

## Background

Population Health Management (PHM) refers to large-scale transformations required for the reorganisation and integration of services across public health, health care, social care and community services in order to achieve simultaneous improvements in population health, quality of care and reduction in cost growth (Triple Aim (TA)) [1] (Steenkamer B, Drewes HW, Baan CA, Putters K, van Oers H. Reorganising and integrating public health, health care, social care and community services: a theory-based framework for collaborative adaptive health networks to achieve the triple aim. Provisionally accepted for publication). To stimulate PHM, a wide range of stakeholders work together to design place-based initiatives and explore which strategies will not only strengthen connections across different sectors, but also transform how health care is delivered in order to address the full range of health determinants and build more healthier communities [2] (Steenkamer B, Drewes HW, Baan CA, Putters K, van Oers H. Reorganising and integrating public health, health care, social care and community services: a theory-based framework for collaborative adaptive health networks to achieve the triple aim. Provisionally accepted for publication).

It seems likely that the success of place-based initiatives is influenced by the alignment between the expectations of the various stakeholders on how initiatives should be developed and the strategies that the various stakeholder groups intend to implement. Previous research has indicated that stakeholders’ intended strategies are based on prior experiences regarding which strategies worked in which contexts and how and why they worked [3–8]. However, it remains unclear what the differences and similarities in prior experiences of the various stakeholder groups that participate in these place-based initiatives were with regard to PHM development. Nor is it clear what their expectations are with regard to how best to develop PHM. This can be related to the dominant focus of previous research on the impact of strategies and on what factors facilitate or inhibit the development of multi-sector partnerships for health [5, 9–11]. Therefore, this study aims to answer the following research question:

How are expectations and prior experiences of the various stakeholder groups that participate in place-based initiatives associated with stakeholders’ intended strategies to further develop PHM?

Practice leaders and policymakers can use the insights into the differences and similarities between the expectations, experiences and intended strategies of the various stakeholder groups, to influence and shape how initiatives could be further developed. Moreover, insight into stakeholders’ experiences regarding which strategies work in which contexts and how and why they work will add to the theoretical understanding of PHM strategies.

## Methods

This explorative study used realist principles. The hallmark of realist inquiry is its understanding of causality, linking interventions, hereafter referred to as strategies (S), contextual factors (C) mechanisms (M) and outcomes of strategies (O) [3, 4, 6, 12]. These links are the so-called SCMO configurations [3, 4, 7]. From a realist point of view, strategies implemented in a specific situation will change this context due to the resources and opportunities these strategies offer or deduct [4, 12, 13]. Due to this changed context, people will change their reasoning or behaviour, which will influence the outcomes of these strategies [6, 12, 13]. For PHM oriented definitions of ‘strategies’, ‘contexts’, ‘mechanism’, outcomes’, and SCMO configurations, see Table 1*.*

**Table 1:** PHM oriented definitions of realist concepts

PHM Strategy	Refers to the intended plan of action [3, 13, 14]. Intended plans of action attempt to create changes by offering (or deducting) resources or opportunities in a given context [15]. In this study strategies refer to the reorganization and integration of public health, health care, social care and community services including ‘partner’ sectors (e.g. housing, transport), to promote the TA.
Context	Pertains to the ‘backdrop’ of programs [13]. For example, the different multilevel sociocultural, historical, economic, political or relational conditions connected to the development of PHM by PHM initiatives that are also changed as a result of the implemented strategies and, which may cause certain mechanisms to be triggered.
Mechanism	Refers to the generative force that leads to outcomes [14]. It denotes the changes in reasoning or behaviour of the various stakeholders (e.g. feelings of multi-disciplinary accountability triggered by the introduction of new financial incentives). Strategies should not be mistaken for mechanisms. Whereas strategies are the intended plans of action, mechanisms are the responses to the intentional resources that are offered [13].
Outcome	Pertains to intended or unintended outcomes of strategies [13]. In this study, the reported outcomes are the measured outcomes as stated in the studies included in this review, e.g. changes in knowledge or new financial arrangements.
SCMO configurations	SCMOs are heuristics that portray the relationships between strategies, context, mechanism, and outcome [3, 4, 7]. The SCMO configurations in the current study present the relationships between the strategies for PHM that, when implemented in a specific context, triggers mechanisms to cause certain outcomes.
Intended PHM strategies	Refers to strategies based on stakeholders’ expectations and prior experiences regarding which strategies work and how and why they work; i.e. the relationships between strategies-contexts-mechanism-outcomes (SCMO configurations).

The process steps within this study were as follows: 1. Identifying the expectations with regard to the development of PHM of various stakeholder groups that participate in place-based initiatives; 2. revealing the deeper explanations, i.e. the SCMO configurations, upon which these expectations are based, and, 3. exploring how expectations and prior experiences are associated with intended PHM strategies*.*

### Data collection

Nine Dutch PHM initiatives that together serve over two million people, took part in this research project (*see* Additional file 1*. for details on the Dutch PHM pioneer sites*). To gain maximum insight into the expectations, prior experiences and intended strategies, representatives (executive level) of all stakeholder groups that participated in the steering committee or that were otherwise involved in regional PHM development, were invited to participate in a (face to face) interview. Three persons declined to participate due to logistical reasons and ten persons were included at the request of the initial invitees, e.g. some preferred to be assisted by their staff. Between June 2016 and February 2017, 55 interviews were conducted with 70 stakeholders of nine Dutch PHM initiatives. The interviews were foremost individual interviews conducted face to face except for 3 interviews that were performed via telephone. The 70 stakeholders were part of seven different stakeholder groups: representatives of hospitals (*N* = 16) (including a long-term care organization); primary care groups (*N* = 11), patient representative organizations (*N* = 5), municipalities (*N* = 17 of which 7 aldermen and 1 representative of the Regional Public Health Service), healthcare insurance companies (*N* = 12), local businesses (*N* = 2), and program managers of the nine PHM initiatives (*N* = 7).

In preparation for the interviews, the authors collected documents concerning the vision, mission and ambitions that were stated by the PHM initiatives at the start of the PHM programs. A semi-structured interview guide was used to support the interview process (*see* Additional file 2). The interviews consisted of three steps. *First*, based on the authors’ assumption that as PHM initiatives develop in time, interviewees might have expectations spread over time, interviewees were asked to write down their short (until 2018, 5 years after the start of the PHM initiative); middle- (until 2023, 10 years after the start) and long-term (until 2033, 20 years after the start) expectations with regard to the development of PHM in a specific data extraction form. *Second*, the expectations were then discussed by asking interviewees to focus on explanations underlying their expectations. These explanations, which were based on prior experiences of what strategies had worked, how and why (i.e. the contextual factors and mechanisms by which these strategies operated), were first asked using open questions. Next, a document was shown to the interviewees that visualized the theoretical framework for PHM named the Collaborative Adaptive Health Network (CAHN) (Steenkamer B, Drewes HW, Baan CA, Putters K, van Oers H. Reorganising and integrating public health, health care, social care and community services: a theory-based framework for collaborative adaptive health networks to achieve the triple aim. Provisionally accepted for publication). CAHN summarizes the insights into how and why PHM can successfully be developed. CAHN describes eight components (*Relation, Social forces, Accountability, Leadership, Resources, Finance, Regulations and Market*) that contain the insights into the relationships between PHM strategies, their outcomes, and the contextual factors and mechanisms that explain how and why these outcomes were reached, and the theories underling these relationships. This document was used to discuss any additional prior experiences underlying the expectations regarding the development of the PHM initiative that were not put forward in the first instance (see second step in which the authors used an open question to gain insight into the prior experiences). *Third*, interviewees were asked for their intended strategies. The researchers discussed with the interviewees how expectations and prior experiences were associated with these strategies.

### Analyses, synthesis and interpretation of the data

The semi-structured interviews containing the expectations, prior experiences and intended strategies were transcribed verbatim and this data was analysed using MaxQDA software. In addition, the expectations with regard to the short-, middle- and long-term period that interviewees had written down in the data-extraction form, were put into a Microsoft Excel sheet and structured along the three time periods, stakeholder groups and PHM initiatives. As the expectations were formulated from the perspective of 70 interviewees and varied on a detail level, they were clustered on the basis of recognition of similarities to ensure richness of data and broad representation of perspectives [16]. Structuring the data in this way, enabled identification of which expectations were limited to one or more time periods, and which expectations were mentioned by the majority of the stakeholder groups and PHM initiatives. Since the 70 interviewees were not equally distributed among the stakeholder groups in terms of numbers, the expectations that were mentioned by a majority of stakeholder groups (at least 4 out of 7) involving a majority of the PHM initiatives (at least 5 out of 9), were included in the further analysis process to ensure sufficient generic validity. As there was a very limited number of perspectives that were shared by less than half of the stakeholder groups, almost all the different perspectives of the stakeholders of the PHM initiatives were included in MaxQDA. Using MaxQDA, these included expectations and their prior experiences (i.e. the relationships between strategies-contexts-mechanism-outcomes (SCMO)) and the intended strategies that were related to these expectations, were identified. By relating the expectations, the prior experiences and intended strategies in an integrated way, themes emerged. Subsequently, for each theme the expectations and underlying prior experiences and intended strategies were put into a Microsoft Excel sheet per stakeholder group. This overview enabled insight into the range and variations per expectation and in the prior experiences and intended strategies within and between stakeholder groups.

### Ethics approval

Approval and consent for this study was provided by the Ethical Review Committee at Tilburg University (EC-2016.27).

## Results

This study identified four overarching themes with regard to the expectations, prior experiences and intended PHM strategies. These themes were: 1. Regional collaboration as a basis for PHM; 2. Governance structures and stakeholder roles; 3. Learning environments that stimulate PHM, and 4. Financial and regulative conditions that stimulate PHM. The themes were intertwined and were represented in each time period. However, short-term expectations were mainly represented within theme 1. Theme 2 and 3 also highlighted short-term expectations, but middle term expectations were more prominent. Expectations within theme 4 mostly represented the middle- and long-term. With regard to the intended PHM strategies, most intended strategies related to the short-term, and no intended PHM strategies were mentioned in relation to the long-term expectations.

Per theme, the variations within and between stakeholder groups’ expectations are described, including the time frame (short-middle-long term). In addition, under the headings ‘prior experiences’ and ‘intended strategies’ per theme is described how the contexts of the various stakeholder groups influenced their reasoning (the mechanisms), and thus the outcomes of prior implemented strategies, and how this influenced the outlined intended PHM strategies of the various stakeholder groups. Furthermore, per theme, reference is made to the respective Tables 2, 3, 4 and 5. The top row of Tables 2, 3, 4 and 5 provides insight into the expectations and intended strategies of the various stakeholder groups participating in PHM initiatives. In the bottom row of each table, the prior experiences on what strategies reached which outcomes (strategies-outcomes), how and why these outcomes were reached (context-mechanism) are described.

**Table 2:** Regional collaboration as a basis for Population Health Management: expectations, intended PHM strategies and prior experiences as reported by stakeholders

Stakeholder groups’*expectation (short (5-), middle (10-), long (20 years) term)		**Stakeholder groups’ intended strategies** (short (5-), middle (10-), long (20 years) term)
Short	**HCI, M, PCG, PM:** Increased collaboration across sectors with an increasing number of stakeholders with an increasing number of target groups. **H, HCI:** Increased collaboration within the care sector on specific population groups. Value for money is established. **B, H, HCI, M, PCG, PM, PRO:** Collaboration is increasingly based on a regional vision - **M, PRO, B:** and increasingly based on (above) regional coordination mechanisms from a social-economic perspective.	**HCI:** Main focus on care sector. Sharpen hospital profiles by allocation, substitution and concentration of specific care. Slowly increase collaboration with municipalities. **H:** Keep complex care in hospital. Delay the shift of low complex care. **HCI:** Investments in regional relationships. Intensify collaboration with municipalities. **B, M, HCI, PCG, PM, PRO:** Investments in shared responsibility based on a long-term vision – data and funding that support an integral policy (**H, HCI, PCG, PM**) - from a social-economic perspective (**B, M, PRO).**
Middle	**HCI, M, PCG, PM: C**ollaboration across sectors with an increasing number of stakeholders with an increasing number of target groups continuous. **M, HCI, PCG, PM:** Increased collaboration between municipalities and healthcare insurers. Shift from curative to preventive care and self-management. **B, HCI, M, PCG, PRO:** Stabilization of decentralization** via sustainable collaboration between regional stakeholders.	**HCI:** idem short term. **PCG:** Expand collaboration with hospitals on current projects and in Public Private Partnerships and with other stakeholders in social sector. **H, HCI, M, PCG, PM, PRO:** Organize larger projects and projects that have more impact on TA, more prevention, more stakeholders using concepts such as Positive Health.
Long	**B, H, HCI, M, PCG, PM, PRO:** Regional health policy is based on a regional vision.	**–**
**Prior strategies and outcomes**		**contextual factors-mechanisms**
**HCI**: Investments in PHM initiatives. PHM is too costly and time consuming. **HCI**: Investments in regional relationships in order to establish regional responsibility for addressing the social determinants of health. Positive experiences. **M:** Collaboration with healthcare insurers for risk groups. Difficulties with establishing business cases. Slow progress. **H:** Mergers of hospitals. Mergers continued. **PCG: -**Substitution of care and professionalizing of PCG organizations. Slow progress. -PCG pacts to influence politics in order to cut hospital budgets, were unsuccessful.		**HCI**: Hindering factors for investments in PHM are highly competitive markets, to many involved stakeholders, too little regional market power of the insurer, no collaborative agenda especially with municipalities, no insight into data to support business cases**. B, HCI, M, PCG, PM:** Hindering factors for PHM are top down management culture within healthcare insurer, differences in legitimacy between healthcare insurer and municipalities***, differences in financial interests, differences in culture (e.g. decision-making structure), differences in operational scale (too small numbers of insured people within one municipality), and high turnovers within healthcare insurers which prohibits understanding the regional situation. **B, M, PCG, PM:** Municipalities have more freedom to invest in projects when purchasing from the Social Support Act, the Participation Act and Youth Aid, compared to healthcare insurers when purchasing from the Healthcare law, which allowed healthcare insurers to only compensate prevention for patients with health problems to prevent worse. **H, HCI:** Business cases are drivers for collaboration. **M**: The healthcare insurers have commercial interests, which municipalities have not. **H:** hospitals experience difficulties regarding the induced 0% growth by the government, high market competition, the demand for more transparency, quality and efficiency, continuous pressures to match supply and demand, financial bottlenecks (i.e. real estate problems), internal resistance to concentration, redistribution and substitution of care. The preconditions of hospital directors’ and MSBs**** to get agreement on a new hospital profile, which healthcare insurers demand, are: more focus, time and upfront financial guarantees. **HCI**. PHM development which is assigned to specific managers within several healthcare insurance organisations facilitated going beyond care. Investments in providers and municipalities are important to address the social determinants of health. **B, HCI, M, PCG, PRO:** Relationships and regional coordination of PHM are the drivers for collaboration. **PCG, PM:** Increased tasks and paperwork do not weigh up to financial uncertainties. Hospitals are too internally focused. Rigorous cuts in hospital budgets are necessary for real transition and real responsibility of healthcare insurers to control hospital budgets. Political pressure on gatekeeper function during the national elections has made PCGs more aware that building on and PHM experiences and showing results was pivotal for their profession.

*B = Businesses; H = Hospital; HCI = Health care insurer; M = Municipality; PM = Program manager; PCG = Physician care group; PRO = Patient representative organization

**Decentralization of tasks from the central to the local government (since 2015) entails safeguarding 1. the wellness of children up to 18 years, 2. the support people need to be able to work, 3. the care and social support people need to live in their own homes as long as possible

***Insurers’ legitimacy: ensure public interests: quality, affordability and accessibility of care to safeguard the macro care-budget and safety and quality norms; municipality’s legitimacy: ensure interest of regional societal issues

****MSBs = Associations of medical specialists within a hospital

**Table 3:** Governance structures and roles: expectations, intended Population Health Management strategies and prior experiences as reported by stakeholders

Stakeholder groups’*expectation (short (5-), middle (10-) long (20 years) term)		**Stakeholder groups’ intended strategies** (short (5-), middle (10-), long (20 years) term)
Short	**B, H, HCI, M, PCG, PM, PRO:** Decreased role of individual local and regional organizations, a regional governance structure is increasingly seen as the right scale for regional responsibility. Increasingly other regional stakeholders will enter. Governance structures will continuously change. Structures will range from informal to formal collaborative networks (on specific subjects). **H, HCI, PCG, PRO**: Different ideas on who will play a lead role. **B, H, M, PM**. Citizens solve local problems (e.g. loneliness) as co-creators with support of professionals. **H, HCI, PCG, PM, PRO:** Increased citizens’ awareness of responsibility for their own health via big data-technologies. The role of ‘co-creative citizens’ is increasingly anchored in the regional healthcare policy (**H, HCI, PCG, PM, PRO**) (strategic, tactical, operational) (**PRO).**	**H, HCI**.: Invest in hospital learning networks. **H, HCI, PCG:** Invest in the bundling of high complex care in hospital networks and low complex care in multidisciplinary centres. Invest in the development of regional governance structures. **B, H, HCI, M, PCG, PM, PRO:** Develop meaningful engagement of citizens. **HCI, M, PCG, PRO: I**nventory of citizens’- patient’s wishes and needs regarding regional health and wellbeing **H, M, PCG, PM, PRO:** Establish a citizens’ cooperative. **B, HCI, M, PCG, PM, PRO:** Activate community building so citizens can self-manage.
Middle	**HCI, PCG:** The beginning of ACO Dutch style. **H, HCI, PM, PCG:** PHM networks are responsible for regional PHM **H, HCI, PM, PCG:** The community is more in the lead. **B, M, PCG, PM, PRO:** A bigger role for municipalities in directing regional health care, while the role of the healthcare insurers is expected to decrease as it insufficiently fits the transformation movement. Citizens are co-creators in the regional healthcare policy.	**H, HCI:** Idem short term. **H, HCI, PCG: I**dem short term. **B, H, HCI, M, PCG, PM, PRO: I**dem short term. **HCI, M, PCG, PRO:** Ensure citizens-patients are co-creators. **B, HCI, M, PCG, PM, PRO: I**dem short-term.
Long	**HCI, PCG, PM:** Accountable Care Organisation – Health Management Organisations.	**–**
**Prior strategies and outcomes**		**contextual factors-mechanisms**
**HCI, H:** Investments in sharper profiles. Hospitals take matters more and more into their own hands. **PCG:** Exert upward and outward influence. In some areas regional agendas were increasingly coupled. **B, H, HCI, M, PCG, PM, PRO:** -Make patient representative organizations part of the PHM governance structure. Limited patient influence was noticed. **-**Organise that citizens take co-director-producer roles of social initiatives. Citizens are increasingly active in the public domain.		**HCI, H:** Technological developments will build organizational power for hospital networks. **H:** primary care groups might be marginalized as they lack professional capacity and knowledge. Hospitals are capable of taking the integrator role. **PCG:** Agendas have become increasingly ambitious by engaging local, regional influential stakeholders and national policymakers. Integrator role is seen as a powerful strategy to influence future governance structure. **B, H, HCI, M, PCG, PM, PRO: -**Stakeholders were in doubt if this organizational representation equalized the representation of citizens. Also, questions remained with regard to what governance structures would be appropriate for patient-citizens engagement. -The political-social relations between the government, the market and the community are changing. Engagement of citizens is necessary to ensure that services are being arranged according to their needs. **B, M, PCG, PM, PRO:** Municipalities are obliged by law to support that more people participate and find work (also people with an occupational disability in collaboration with regional businesses), and to support citizens to arrange matters themselves in the public domain (‘Do-democracy’). Democratization and decentralization will erode healthcare insurers’ role in time.

*B = Businesses; H = Hospital; HCI = Health care insurer; M = Municipality; PM = Program manager; PCG = Physician care group; PRO = Patient representative organization

**Table 4:** Learning environments that stimulate Population Health Management: expectations, intended PHM strategies and prior experiences as reported by stakeholders

Stakeholder groups’*expectation (short (5-), middle (10-), long (20 years) term)		**Stakeholder groups’ intended strategies** (short (5-), middle (10-), long (20 years) term)
Short	**H, HCI, M, PCG, PM: L**earning environment are being developed. **H, HCI:** Learning hospital networks will be established. **B, HCI, M, PCG, PM:** Municipalities and healthcare insurers will more and more exchange data and share purchase knowledge and expertise to gain insight into costs and benefits. **PCG, PM, PRO:** Start of regional IT structure.	**H:** Invest in technological developments, education, knowledge and employment of staff and in creating and strengthening an innovation culture. **B, H, HCI, M, PCG, PM, PRO:** Invest in technological means and training. Provide insight into needs, quality and costs for clear decision support. Investigate what indicators and data are needed for Value Based Health Care and Positive Health. **HCI, PCG, PRO:** Invest in business cases and the Plan-Do-Study-Act cycle at all levels. Invest in value for money, i.e. by linking Patient-Reported Outcome MeasureS to data, introducing nudging (e.g. care-miles).
Middle	**H, HCI, PCG, PM, M:** increase in E-health, personalized health and start of patient-ownership of health files. More care is delivered closer to home with use of technology. Patients have an active role in shared decision-making based on data. **H, PCG, PM:** Technology will lead to a higher demand for technical staff and a need for other competences and training. Staffing will be a challenge.	**H:** Appoint an intermediate between the user of technology and the supplier of technology. **H, HCI, PCG, PM**: Organize patient ownership of health files and technical devices.
Long	**PRO, PM, H, PCG:** Technology has changed professionals’ and patients’ roles. Regional health policy is based on population data and matching financial arrangements. National IT structure	**–**
**Prior strategies and outcomes**		**contextual factors-mechanisms**
**H, HCI, M, PCG, PM:** low investments in technology. Investments are just enough to meet the requirements of electronic patient files, quality records and the existing method of financing. **H, HCI, M, PCG:** Efforts to share data. This is difficult within initiatives, especially when 2/more healthcare insurers take part or between healthcare insurers and municipalities. **H, PCG, PRO:** Stimulation of more insight into health records and needs, costs and quality of care and support. This subject is high on the agenda of the public.		**HCI, PCG, H, PM:** Organizations work on timely and targeted feedback to providers and administrators. Organizations increasingly understand that this can contribute to insight into the demand and needs of the population, quality of care, and cost-effectiveness and to the willingness to choose the best treatment-support at the lowest price, to innovate consistently and to organize (long-term) financial arrangements. **H:** investments in technology are necessity to achieve a shaper hospital profile. **B, H, HCI, M, PCG, PM:** The data-technology lacks behind the desired information need, which induced tenseness. **H, HCI, PCG:** for hospitals investments in technology were key. Hospitals were reluctant to share data with primary care groups and healthcare insurers as this could influence their financial budget. **HCI:** Some organizations are reluctant to share cost data with the healthcare insurer because opening their books will set back their bargaining power. Continuous leadership support is important when sharing data to support a learning environment. **M, HCI:** lack of insight into data produced tensions between municipalities and healthcare insurers. **H, HCI, PCG:** lack of clarity on regulative restrictions on specific types of data-sharing between healthcare insurers, hospitals, primary care groups and between health care insurers. **B, HCI**, **M, PCG, PM, PRO:** Care and support is increasingly planned around patients. Organizations are more aware that, in principle, patients or their family have control. In addition, as citizens-patients are co-creators of their own health, insight into health records and needs, and the quality and costs of prevention, care and community services is necessary to enable this co-creatorship. The influence of citizens-patients will increasingly be supported by modern technology. **B, H, HCI**, **M, PCG, PM, PRO:** The real upheaval in healthcare will only take place if patients increasingly use this technology.

*B = Businesses; H = Hospital; HCI = Health care insurer; M = Municipality; PM = Program manager; PCG = Physician care group; PRO = Patient representative organization

**Table 5:** Financial and regulative conditions that stimulate Population Health Management: expectations, intended PHM strategies and prior experiences, as reported by stakeholders

Stakeholder groups’*expectation (short (5-), middle (10-), long (20 years) term)		**Stakeholder groups’ intended strategies** (short (5-), middle (10-), long (20 years) term)
Short	**B, HCI, M, PCG, PM, PRO:** No changes in the finance system, certainly no payment models for the total population as originally planned. First TA results will be achieved on intervention level. Business cases based on the TA model. **B, M, PCG, PRO.** First TA results on intervention level. Shared savings as incentives on an increasing number of projects; first experiences with regional budgets. **PM, PCG, HCI, H:** The purchasing procedures will change. **H, HCI, PCG, PM:** Regulations restricting the data-sharing will not be changed shortly.	**H, HCI, PCG:** Organize multi-year contracts. **H, HCI:** Invest in business cases based on value-based health care. Integral payment model for mental health care, frail elderly and birth care, **HCI, PCG, PM, PRO:** Invest in incentives such as shared savings and use revenues for investments in the PHM initiative. **PCG, PM:** Determine the purchasing process together with the health care insurers and providers and pay more attention to prevention: **HCI, M, PCG:** Pull funds together for specific interventions for specific populations in light of positive health in specific neighborhoods.
Middle	**B, M, PCG, PM, PRO:** Bundling of budgets across sectors-TA outcomes for the whole regional population. Regulations are changed for closer collaboration, combining budgets, payment model for the total population. **H:** Payment of complete pathways instead of payment of separate parts of the pathway. **B, H, HCI, M, PCG, PM, PRO:** Rules are changed for data sharing	**HCI:** Experiment with subscription fees that are in line with the practices’ population, combined with a bonus on outcomes that are of joint interest to the entire population. **H, HCI, PCG, PM, PRO:** Keep experimenting with data optimization. **HCI, PCG, PM, PRO:** Engage politicians.
Long	**B, H, M, PCG, PM, PRO:** Citizens’ coordination of regional health’ financial arrangements. **B, H, HCI, M, PCG, PM, PRO:** Regional health policy is based on big data with matching financial arrangements.	**–**
**Prior strategies and outcomes**		**contextual factors-mechanisms**
**HCI:** Investments in multi-year contracts with hospitals to reduce volume and costs of care. Shared savings incentives for specific projects. Resistance to outcome funding and new payment models and shared savings agreements based on the total population of the PHM initiative. **B, H, HCI, M, PCG, PM, PRO:** Improve efficiency and quality motivated by financial incentives. Business cases that are positive from a societal perspective but negative from an organizational perspective are a problem. **PHCI, M, PCG:** Exchange of data to develop business cases for PHM development. This has challenged the purchasing procedures. Exchange of data sensitive to competition between healthcare insurers is prohibited.		**HCI:** Hospitals received budget guarantees via multi-year contracts to adjust the company for substitution of care to primary care groups. Contracts could be brokered if the quality of care was reduced and requirements were included within contracts, e.g. to cooperate in data-infrastructure development. Furthermore, no savings incentives for the total population were made due to lack of upfront financial investments, lack of data and knowledge to measure total population’ effects, and insurers did nor prefer interference of an integrator needed to divide the savings. Limited experience with alternative ways of payment. Insurers did not prefer outcome payment due to the danger of patient selection. No preference for region wide population payment due to fear of a shift in responsibility to an integrator. Insurers feared that shifting accountability to providers would increase the information asymmetry in favor of providers, and would lead to loss of control over providers, and weaken their purchasing power. **B, H, HCI, M, PCG, PM, PRO:** Leadership and trust are preconditions for financial experiments. Fragmented financing and market forces inhibit structural change. **H, HCI, PM, PCG:** Current policy and purchasing process cannot guarantee efficiency and affordability, accessibility of care and support. The NZa** sets the payment infrastructure, however rational business cases sometimes do not fit into the system, then the NZa should redefine payment structures. Also, the market in which providers have to compete does not fit their need to collaborate for PHM. **PCG, PM:** Budgets allocated to specific compartments such as hospital care within the budgetary framework of the government, hinder substitution of secondary care to primary care. **B, HCI, PCG, PM, PRO:** The Competition Act (ACM ***rules) has rules on data exchange between stakeholders in light of maintaining a level playground. Market competition and payments must be based on health gains. However, the privacy legislation is about privacy protection but not about care optimization. The question is whether it is not the other way around: is it not against the law to not use possibilities that exist for optimization of care, as the law on the medical treatment contract (WBGO) says that professional should present the best treatment to patients. Rigorous changes are necessary in the payment system, legislation and regulations for true transitions in health care. Professionals have experienced that confidence and experimental space and an upfront guarantee that their actions are in line with the legal frameworks or are permitted by supervising organization(s), is necessary.

*B = Businesses; H = Hospital; HCI = Health care insurer; M = Municipality; PM = Program manager; PCG = Physician care group; PRO = Patient representative organization

**NZa: The NZa establishes descriptions of the treatments (performance, e.g. maximum rates), and supervises healthcare providers and healthcare insurers

***ACM: The Dutch Authority for Consumers and Markets is a Dutch independent public regulator charged with the supervision of competition, telecommunication and consumer law

## Regional collaboration

### Expectations

Overall, the majority of stakeholder groups expected an increase in regional collaboration via expansion of target groups within the PHM program (e.g. youth, frail elderly and people with mental health problems) with an increasing number of stakeholder organizations and sectors (e.g. care and nursing homes, home care, municipalities and businesses) in the upcoming years. (*see* Table 2*.*)*.* In addition, stakeholders expected a regional health policy that was based on a regional vision that integrated health with other domains (e.g. education, housing, economics) in the long-term (2033) to support the development of a healthy, vital and economic thriving region.

### Prior experiences

This study identified that stakeholder groups’ prior experiences differed the most within healthcare insurers, between healthcare insurers and municipalities, and between hospitals and primary care groups (*see* Table 2)*.* With regard to the healthcare insurers, at one extreme, this stakeholder group had encountered negative experiences with pushing PHM in contexts they in hindsight regarded as too complex and which jeopardized their control over the purchasing process and their wish to establish value for money. For instance, PHM initiatives within highly competitive markets, and with PHM governance structures containing many different providers and other payers, were considered strong inhibitors for the development of PHM. This was due to e.g. the difficulty of aligning interests of all involved stakeholders. At the other extreme, healthcare insurers, specifically in those areas in which they had a dominant market position, had experienced that investments in regional relationships with municipalities, regional providers, businesses and educational institutions, were strong driver for regional collaboration. In addition, although they had experienced that positive business cases were important, these were more and more viewed from the perspective that in order to address the social determinants of health, which they acknowledged as being strong drivers for health, regional collaboration was necessary.

With regard to municipalities, interviewees indicated that due to the decentralization of tasks from the central to the local government in 2015 (*see* Table 2.), the role of municipalities on regional health and social issues had increased, which was increasingly reflected in the PHM initiatives. Interviewees experienced differences in how municipalities and healthcare insurers approached PHM development within initiatives. Municipalities, program managers, businesses and primary care groups stated that municipalities’ contexts as local governments offered for instance much more latitude to invest in a healthy and prosperous region than healthcare insurers due to e.g. differences in decision-making processes in the purchase of healthcare.

*If we believe in a project, then politically we can move much faster than health care insurers, no cost-benefit analysis but much more if it’s good then we’ll invest in it. Also, we as a municipality are much more aware of what is happening in our region, we know the people. [ … ] For us as a municipality it has to do with seeing the connection between health, education, employment, participation, and to connect the citizens themselves to the higher goal of better health and vitality* (Social innovation official Municipality; I26)*.*

In addition, particularly municipalities, and business but also healthcare insurers that focused on regional relationships, primary care groups including program managers and patient representative organizations situated in economic less prosperous regions had experienced that to establish a vital and economic competitive region, strategies had to be based on a social and economic perspective. Hospitals and primary care groups had experienced uncertainties surrounding the national budgetary boundaries (zero growth for hospitals and a small growth for primary care) set by the National government in 2016. In addition, hospitals had experienced internal and external pressures on the hospital market (*see* Table 2.). These pressures negatively influenced progression on substitution of care to primary care groups within the PHM initiatives. Interviewees stated that this was mostly due to the high priority within hospitals towards the inner organization and perceived financial risks (*see also Themes 2, 3, 4*). Furthermore, at the time of interviewing, primary care groups had experienced political pressures in the run-up to the national elections for a new government in 2016, as political parties discussed several new options regarding the organization of healthcare and the position of providers including diminishing the gatekeeper function of general practitioners.

### Intended strategies

This study identified that although all stakeholders expected an increase in regional collaboration within PHM initiatives, prior experiences with regional collaboration influenced the focus and speed of the intended collaborative strategies. As a result, the intended strategies differed between healthcare insurers, municipalities’ and providers. The focus of healthcare insurers at the one extreme was to stay close to ‘the business of care’, using positive business cases underlying multiyear contracts preferably with organizations where the most value for money could be reached such as hospitals or mental health care organizations. In addition, they intended to slowly organize collaboration with municipalities in small scale interventions with sufficient return on investments in the short and middle term. The healthcare insurer at the other extreme intended to increasingly invest in preventive activities e.g. in larger neighbourhoods or regional projects that would have more impact on TA outcomes for the population in the next years, using concepts such as ‘positive health’ [17], as instruments to success.

*[ … ] and we as a health care insurer, want to demonstrate our added social value. Investing in developments such as ‘​​Positive Health’ and ‘Value Based Health Care’ are important to determine this added value. We are working hard on this to see how quality can be defined differently, like happiness and well-being of people. [ … ] Therefore, collaboration with municipalities is becoming more intense because you have to be careful that you do not throw your problems over the wall* (CEO Healthcare insurer; I16).

Municipalities stated that, supported by the decentralization movement, they intended to (further) develop PHM in order to focus and invest in a healthy, vital and economic competitive region together with healthcare insurers, providers, regional businesses and educational institutions. With regard to collaboration between hospitals and primary care groups, the financial uncertainties mostly influenced hospitals’ strategies towards substitution of care on the short term. Interviewees stated that hospitals intended to obtain sufficient financial-contractual latitude with healthcare insurers to develop a new and sharper hospital profile, and in the meantime delay the shift of (low) complex care until more financial certainty and more certainty about the regional spread of specializations and planning of tasks and personnel within the regional hospital sector was reached. Meanwhile, primary care groups saw the importance of building on the experiences in the PHM initiatives so far and safeguarding the position of primary care i.e. the gatekeeping function of general practitioners in the future. Therefore, they focused on a two-pronged strategy. First of all, primary care groups intended to expand their collaboration with hospitals on current and new patient groups by investing in ways that were of interest from an entrepreneurial perspective as well as from a medical developmental perspective, such as setting up Public Private Partnerships around new medical technological developments. Second, primary care groups intended to expand their PHM strategies toward stakeholders within the social domain. The upcoming concept ‘positive health’ was viewed as a good starting point.

*[ … ] if everybody would consider the social determinants of health, then I expect that the majority of what we now see in the physician practices has nothing to do with care. It has to do with poverty, not having a job [ … ]* (Executive physician care group; I28)

## Governance structures and stakeholder roles

### Expectations

All stakeholder groups expected a decrease of the role of individual organisations in the near future and envisioned that the collaborative of stakeholders within PHM initiatives would eventually carry full regional responsibility for the health and well-being of the total regional population by 2033 (*see* Table 3.). In addition, stakeholders expected that PHM initiatives would continue to adapt their governance structures to fit this regional responsibility. Hospitals, healthcare insurers and primary care groups expected highly complex healthcare to be distributed across hospital networks, and low complex hospital care to be bundled in multidisciplinary centres within Health Management Organisation (HMO) – Accountable Care Organisation (ACO) structures (2033). Most hospitals and half of the primary care groups expected to play a leading role in PHM. In addition, all stakeholder groups expected that the engagement of citizens and patients in the governance structure of the PHM initiatives and its participating organizations would ensure the needs of the regional population in the future.

### Prior experiences

In recent years, hospitals increasingly had to counter financial cuts in overhead. According to representatives of hospitals one of the consequences was that their main focus had been to increasingly bundle knowledge, technological investments and organizational power in hospital networks and public private partnerships (*see* Table 3*.*). They had experienced that these developments, in combination with technological developments, already had led to more cooperation between hospitals and physician care groups. However, with regard to the latter the majority of hospitals stated that physician care groups lacked in professionalizing their policy and management activities to fit their new role related to substitution of care such as providing sufficient GPs with expertise in a specific sub-specialism.

*[ … ] the technological developments for the large group of chronic patients will lead to a 40% decrease in the total of outpatient visits [...] Maybe this will lead to even more collaboration with general practitioners but it could also lead to the erosion of care as the ‘gatekeeper’ function could for the most part be taken over by software devices, and it is questionable if primary care groups are capable of developing beyond a facility company for general practitioners, which they currently are. [ … ] These technological developments and our organisational power could work to our advantage with regard to our leading role and could negatively affect the role of primary care groups in the future. (Senior executive hospital; I39).*

Meanwhile, several leading general practitioners of frontrunning primary care groups that participated in PHM initiatives had accomplished influential leading positions within regional executive ‘table of tables’, in which strategic priorities for the region as a whole were discussed. This development had opened up possibilities for expansion towards collaborative health networks, upon which new governance structures could be built in the future.

With regard to the role of patients-citizens on a governance level, earlier experiences with engaging patient representative organizations within the PHM initiatives’ governance structures was limited and according to interviewees had had limited success due to these organizations’ lack of (specific) expertise on a strategic level. The influence of patients-citizens was foremost limited to the operational level, e.g. sharing their experiences and using these as an inspiration for the transformation of health service pathways. At the time of interviewing, in one PHM initiative, stakeholders had recently introduced a citizen’s cooperative to engage citizens in the development of PHM. The idea was that citizens could use this legal entity to influence what care and support will be delivered in the region. The instrument that would make this possible was a regional health insurance policy. However, the legal entity was still in its infancy. Except healthcare insurers, stakeholder groups speculated that if these and other developments regarding the promotion of citizens’ participation as well as the decentralization of tasks from central government to municipalities continued, the role of the healthcare insurer would no longer be needed.

### Intended strategies

Stakeholders’ prior experiences highly influenced their intended strategies. Hospitals intended to continue the chosen path mentioned above and organize high complex care in higher volumes in fewer regional hospital networks and play a leading role in PHM development. In addition, hospitals intended to slowly organize (low) complex care for specific target groups in alignment with regional stakeholders in multi-disciplinary centres. Meanwhile, leaders of frontrunning primary care groups that had experienced that PHM initiatives did not stand on their own but operated in a wider regional transition field, intended to further build upon regional tables towards regional collaborative health network structures.

Also, with regard to engagement of patient and citizens, prior experiences highly influenced stakeholders’ intended strategies. Due to the limited success in engaging patients and citizens based on specific expertise, all stakeholder groups were still thinking about how best to engage citizens in the PHM governance structure. Most stakeholder groups were leaning towards engaging citizens in the role of a more moral authority and not necessarily on the basis of specific expertise.

## Regional learning environments

### Expectations

All stakeholders expected that the development of a learning environment would go along with the incremental development of PHM (*see* Table 4*.*). In addition, all stakeholders expected that the real transition in the health system will take place due to patients’- citizens’ increased use of technology, as a result of which providers need training and knowledge to coach patients and provide them with good, objective information in order to decide on the most optimal treatment.

### Prior experiences

Interviewees from all stakeholder groups indicated that they had gained experiences in setting up a data-infrastructure, in training healthcare professionals in the use of the data-infrastructure and in giving timely and targeted feedback to individual care providers and administrators in order to support the operationalization and implementation of new interventions (*see* Table 4*.*). This had contributed to more awareness and willingness to change how and what care is offered, and to experiments in shared decision-making based on real time data. However, according to interviewees, the IT developments still lagged behind the desired information needs needed to take further steps towards PHM. At the time of the interviews, this had led to tensions between hospitals, primary care groups and healthcare insurers, between municipalities and healthcare insurers and between healthcare insurers themselves. Stakeholders indicated these tensions were associated with contextual factors such as securement of (financial) interests (hospitals), inability – hesitation to give insight into the necessary data upon which business cases surrounding substitution of care could be built (healthcare insurers), lack of knowledge or consensus on which data – indicators were needed to support the transformation of health pathways (healthcare insurers, hospitals, physician care groups), and lack of clarity about what is legally permitted regarding linkage of data (healthcare insurers, hospitals, physician care groups) (*see theme 4*).

*Quality is what we need, comparing data, benchmarking and not the resistance of professionals [...], the fear organizations have that their interests may be at stake, and that we deal with them in such a clumsy way that they get away with it and maybe if we are not careful they will get away with it in the next five years. These are the real problems.* (Innovation manager healthcare insurer; I54)

### Intended strategies

Because hospitals needed to secure their (financial) interest (*see themes 1, 2 and 4*), their main focus was to further invest in technological developments and specialization and planning of medical technical staff in the short and middle term, in order to sharpen their profile and realize specific person-centred network care within hospital networks. The other stakeholder groups indicated that despite the experienced difficulties mentioned above, they intended to further invest in gaining insight into supply and demand, quality and costs of prevention, care and welfare, as they realized this was essential for establishing continuous improvements. However, primary care groups in particular stressed that knowledge and support for instance from knowledge institutions, clarity from the government about the linking of data (*see theme 4*) and a financial reward for care providers for the delivery of meaningful data, were necessary to realize a learning environment.

## Financial and regulative conditions that suit the stimulation of PHM

### Expectations

All stakeholder groups expected changes in the middle- and long-term with regard to the current financial system, laws and regulations, and accountability procedures that would stimulate improvements in the TA (*see* Table 5*.*). All stakeholders expected that between 2023 and 2033 the current funding and payment models within the health care system would be replaced by other models. However, while healthcare insurers were more cautious with regard to their expectations of a particular model, the other stakeholder groups disagreed about which model was most suitable for realizing the TA: payment models for the total regional population, payment per (care) activity with shared savings – bonuses, or integral payment models. In addition, stakeholders expected changes in laws and regulations for organizations to: 1. Work more closely together without changing the freedom of choice of providers; 2. To share data; and 3. To combine budgets across sectors, or to be held responsible for the health of the total population.

*If you really want to take steps in substitution, the Ministry of Health, Welfare and Sport has to change their policy by rigorously removing money from the hospitals and partly allocating this to primary care. Currently, no one dares to take the lead because they don’t want to risk their reputation* (CEO physician care group: I2).

### Prior experiences

Healthcare insurers indicated that their cautions towards new forms of payment and funding were based on a lack of experiments in the past in alternative payment models in which questions such as what and how much risk organizations could take or which outcome measures would be most suitable, were addressed (*see* Table 5.). In addition, the healthcare insurers that intended to stay close to ‘the business of care’ (*see theme 1.*), believed that new ways of payment and funding would increase information asymmetry, which would imply shifts in accountability to providers that could result in loss of control over the providers and weaken healthcare insurers’ purchasing process. Furthermore, all stakeholders had experienced that leadership and trust were necessary conditions for experimenting with new forms of payment and funding. Stakeholders indicated they had been able to build trusted relationships in the last 5 years since the PHM initiatives had started and during which the first positive results on the TA for specific interventions and subpopulations were achieved. However, during this period of time, leaders of stakeholder organizations ran into issues that hampered the development of PHM, such as restrictions on data sharing (see theme 3), the lack of invoicing codes for new types of services, and the way budgets within the national budget framework are distributed, which hindered the substitution of care. Additional questions that also needed answers were for example how to take financial risks for the total health care costs of the regional population and how to compete while at the same time cooperate between organizations without risking loss of freedom of choice for patients.

### Intended strategies

Although stakeholders were of the opinion that the current payment and funding models did not sufficiently stimulate simultaneous improvement in the TA, they (i.e. primary care groups, businesses, and patient representative organizations), intended to continue to organize care and support in a more coherent way to impact the full range of health determinants, as they hopefully expected that payment models and funding will be adjusted in the middle-term. Meanwhile, intended strategies between healthcare insurers differed. The healthcare insurer that predominantly intended to ‘stay close to the business of care’, primarily focused on providing multi-year contracts to hospitals that showed trusted leadership, clinical responsibility and which were able to achieve healthy financial conditions. In addition, they intended to stay in control of the purchasing process by investing in continuous monitoring to prevent information asymmetry. Furthermore, they intended to experiment in integral payment models (e.g. birth care, mental health, frail elderly). Healthcare insurers that predominantly intended to invest in regional relationships and responsibility were, just like municipalities, more focused on experimenting with a combination of models, demonstrating returns on investment, identify ways of overcoming administrative barriers to coverage, align administrative processes and distribute data to stimulate efficiency and evaluation.

In addition, to stimulate changes in laws and regulations, several front running primary care groups’ strategies were aimed at continuously influencing the ministry of Health, Welfare and Sport and national interest groups on subjects that hindered PHM development. Subjects that were put forward were e.g. restrictions in data integration due to the current privacy law (*see theme 3*), municipalities having more latitude than healthcare insurers in organizing business cases that bridged sectors (*also see themes 1, 2*, *3*), and restrictions in substitution of secondary care to primary care due to the current budgetary framework of the government.

## Discussion

This study identified stakeholder groups’ short-, middle- and long-term expectations of PHM development, the underlying explanations for these expectations and their intended strategies. These expectations, their underlying explanations and intended strategies could be categorized into four themes: 1. Regional collaboration as a basis for PHM; 2. Governance structures and stakeholder roles; 3. Learning environments that stimulate PHM, and 4. Financial and regulative conditions that suit PHM. These themes are intertwined. Although stakeholders mostly agreed on long term-overall expectations, the short and middle term expectations and prior experiences largely differed between stakeholder groups and within the stakeholder group healthcare insurers. These differences influenced stakeholders’ intended strategies towards PHM development. Healthcare insurers that highly valued control over the purchasing process and value for money, intended to stay close to the business of care, in comparison to insurers that valued regional relationships in order to establish regional responsibility for health and social issues. The latter were more keen to invest in data-sharing, and in experiments with data-technology, new forms of payment, funding and accountability. Of all providers, hospitals’ strategies were the most internally focused. This internal focus was mostly due to ongoing financial pressures that hindered the shift of low complex care to primary care groups, data-technology development and the sharing of data, and experiments with new forms of payment. Of all stakeholders, municipalities and regional businesses were the most driven to address health and social issues from a socio-economic perspective and on a regional scale in order to establish a vital and economic competitive region. This was mainly based on municipalities’ decentralization tasks and on businesses’ interest to support healthy behaviour of employees.

The current study showed that collaboration between an increasing number of stakeholders and extension of the portfolio of the PHM initiatives were mainly expected in the short term, while more experiments with new payment models and funding were mainly expected in the middle and long term. These results are in line with previous literature, which has shown that specific activities are associated with specific phases in PHM development [18, 19].

As described in theme 1, the way healthcare insurers operationalized their tasks to safeguard the quality, affordability and accessibility of care, influenced how PHM development. These findings are in line with previous literature [20–22]. PHM initiatives in which the healthcare insurers interpreted their role as ‘regional financial manager’ from a relational point of view, could make more progress with regard to the focus and speed of PHM development than PHM initiatives in which the healthcare insurers primarily focused on staying close to the business of care. In addition to previous literature, this study has given insight into the underlying experiences, i.e. the conditions and motivations that influenced the choices of stakeholders’ intended strategies. For instance, the insurers that had had negative experiences in pushing PHM, which had jeopardized their control over the purchasing process, intended to stay close to the business of care as they expected this strategy was the best way to achieve value for money.

The governance structures of the pioneer sites have been adapted over time to guide the development of PHM and all stakeholders expected this trend to continue. However, up until now there is still no clear picture of how the governance structures of PHM initiatives will further evolve and how the roles between the organizations will be divided and who will take responsibility for the total population in the future. This is comparable to place-based initiatives in for instance the United States, the United Kingdom, Canada or Germany, which have shown that governance structures are divers and that changes in governance structures have many reasons such as lack of commitment, lack of interest and lack of resources [2, 18, 23]. In addition, in line with previous literature, PHM initiatives do not stand on their own but operate in a wider transition field in which PHM initiatives connect nodes within the network and build upon regional developments towards regional collaborative health network structures [2, 18] (Steenkamer B, De Weger E, Drewes HW, Putters K, van Oers H, Baan CA: Implementing Population Health Management: An international comparative study, submitted for publication).

Moreover, as described in theme two, despite stakeholders’ conviction that involving patients/communities can help ensure that services are more tailored to their needs and thus ultimately improve community health outcomes, all stakeholder groups remained unsure on how to implement more ‘meaningful’ community engagement within their own contexts. These findings are in line with previous literature [24, 25]. Based on the results of this study the discrepancy between the intended strategies and expectation towards future roles could be attributed to prior negative experiences with patient engagement. To engage patients/communities more meaningfully, PHM initiatives could draw inspiration from previous studies (e.g. de Weger et al., 2018). For example, in the Netherlands, some communities and municipalities have been experimenting with involving citizens in the planning and decision-making of how municipalities’ budgets should be spent [26]. The experiences gained in citizens involvement can serve as examples on how communities can be involved in developing a regional health policy based on a shared vision (*Theme 1*), be involved in initiatives’ leadership and management structures (*Theme 2*), in helping to identify citizens’ needs (*Theme 3*) and in setting financial priorities (*Theme 4*).

Moreover, in addition to preconditions such as trust and leadership, investments in data infrastructures and technologies and additional knowledge, expertise and capacity are needed for the introduction of new ways of payment and funding. Alternative payment models seem effective to actually realize the TA, however, these take a long time to iron out and the pros and cons need to be properly monitored to make adjustments possible [20, 21, 27]. In addition, the consequences of technological developments to utilize existing information systems of professionals and citizens are linked to adjustments in privacy- and other laws, and to adjustments in accountability procedures. To further speed up PHM development there is a need for government support such as clarity about data integration and financial support such is the case for the Accountable Health Communities in the United States, that receive resources, including financial support and technical assistance specifically intended for aspects of PHM development such as setting up a learning environment [28]. This could contribute to reducing the tensions stakeholder groups encounter (ed).

This study has several limitations. One being that the information provided by the interviewees is subjective information formulated from the perspective of the stakeholder. In addition, the 70 interviewees were not equally divided over stakeholder groups. Therefore, the analysis was set up to only include the perspectives that emerged in at least half of the stakeholder groups and PHM initiatives. However, as there was a very limited number of perspectives that were shared by less than half the stakeholder groups, this study contains almost all the different perspectives of the stakeholders of the PHM initiatives. In addition, the analysis and synthesis of the data was performed by two researchers and verified by the research team which renders confidence to the reliability of the results. Furthermore, PHM strategies for 2023 were less put forward by interviewees in comparison to those for 2018. For 2033, PHM strategies were lacking completely. This can be explained by the fact that especially for the long-term time frame, stakeholders indicated that from a political and economic perspective 20 years was too unpredictable.

This research contributes to the theoretical understanding of PHM strategies by giving insight into what strategies work and how and why they work. In addition, practice leaders and policymakers can use the insights into the expectations on the future development of PHM of a diverse range of stakeholder groups, their prior experiences and their intended PHM strategies to better stimulate and coordinate PHM development. Future research should investigate how regional financial management can best be executed and what the roles of healthcare insurers, municipalities and third parties (integrators) should be in order to further push PHM, and who best can take responsibility for the health of the total population in the future. In addition, future research should investigate in what way citizens can best be involved in PHM development. Furthermore, it should be investigated how the government and supervising organizations can best stimulate investments in regional learning environment such as data-technology and knowledge-development, and how best to stimulate market-collaboration and new payment models that promote simultaneous improvements in the TA. An example of a program that could be investigated is the Dutch National Program ‘The right care at the right place’, which e.g. provides a regional basic dataset that can help healthcare insurers municipalities and providers in mapping the current and future care and support needs and the current offerings [29]. Moreover, research could further investigate differences in values and convictions of the various stakeholder groups that could hinder PHM.

## Conclusion

The differences in intended strategies between stakeholder groups and within the stakeholder group healthcare insurers were mostly based on differences in prior experiences i.e. specific contextual factors that stakeholders had experienced and that hindered progress in PHM. Barriers that stakeholder groups encountered were related to e.g. differences in values and convictions, information asymmetries which could endanger the purchasing process, lack of insight into data to support business cases or financial uncertainties due to political pressures. These barriers made stakeholders more reluctant to take steps beyond their usual practice and push PHM further. In addition, stakeholders indicated that government support was needed to e.g. reduce barriers between stakeholder groups related to restrictions within laws and regulations such as providing clarity about data integration, market-collaboration and also (financial) support intended for specific aspects of PHM such as new payment models that stimulate PHM, and setting up and improving learning environments. Policymakers and practice leaders can use these insights to reduce these uncertainties and establish more comfort in order for all stakeholder groups to jointly establish PHM.

## Supplementary information

Additional file 1. Characteristics of the nine Dutch PHM initiatives (DOCX 16 kb)

Additional file 2. Interview guideline (DOCX 54 kb)

## Data Availability

The datasets used and analysed during the current study are available from the corresponding author on request.

## Abbreviations

ACO: Accountable Care Organisation
CAHN: Collaborative Adaptive Health Network framework
CEO: Chief Executive Officer
HMO: Health Management Organisation
PHM: Population health Management
SCMO: Strategy-Context-Mechanism-Outcome configuration
TA: Triple Aim
