# Supplementary material for: How executives’ expectations and experiences shape population health management strategies
Source: BMC Health Serv Res. 2019 Oct 26;19:757. doi: 10.1186/s12913-019-4513-3 (PMC6815420; doi:10.1186/s12913-019-4513-3)
Supplement: Supplementary file 1 — Characteristics of the nine Dutch PHM initiatives (DOCX 16 kb) [file 12913_2019_4513_MOESM1_ESM.docx]

**Additional file 1. Characteristics of the nine Dutch PHM initiatives**

| Starting in 2011-2012, PHM initiatives developed to reorganise and integrate public health, health care, social care and community services in various regions in the Netherlands. The aim of these initiatives was to achieve improvements in the Triple Aim. In order to gain insight into and learn from their experiences, the Minister of Health, Welfare and Sport wanted to monitor a number of these regional initiatives. The Minister designated nine regional initiatives, which had been put forward by healthcare insurers, as so-called ‘Pioneer sites’. The initiatives were supported by a National platform to exchange their experiences with the Ministry and supervising institutions. In addition, the initiatives were included in the National Monitor for Population Health Management, which was executed by the National Institute for Public Health and the Environment from 2013 to 2018.  At the start (2014), the core stakeholders within most Pioneer sites were primary care organizations, hospitals, healthcare insurers and patient representative organizations, which in time were supplemented to a varying extent with other stakeholders such as municipalities, businesses, long term care and home care organizations, and educational institutions.  The populations of the Pioneer sites are demarcated in various ways: geographically (all inhabitants of one or more municipalities) or on the basis of the service area of ​​general practitioners affiliated with the physician care group included in the PHM initiative, and finally a distinction can be made between whether or not people are contracted by the involved health care insurer.  At the start the Pioneer sites selected interventions, which were often focused on themes such as substitution, integration of care (in some cases with community services), self-management and prevention. They gradually expanded their PHM program by bridging sectors and by adding new stakeholders and interventions, such as mental health care and youth care.  Since their appointment by the Ministry, the Pioneer sites have undergone a number of changes, both in their governance structures, management, the development of interventions, and in ways of funding and contracting (1-4). |
| --- |

1. Drewes HW, Struijs JN, Baan CA. How the Netherlands is integrating health and community services. NEJM Catalyst. 2016.

2. de Vries EF, Struijs JN, Drewes HW, Heijink R, Baan CA. Key drivers of payment reform in population health management, lessons from nine Dutch pioneer sites. International Journal of integrated Care. 2017;17(5).

3. Hendrikx R, Spreeuwenberg MD, Drewes HW, Ruwaard D, Baan CA. How to evaluate population health? A study of nine population managfement initiatives. International Journal of Integrated Care. 2017;17(5):1/8.

4. Steenkamer B, Baan CA, Putters K, Van Oers H, Drewes HW. Population health management guiding principles to stimulate collaboration and improve pharmaceutical care. Journal of Health Organization and Management. 2018;32 (2).
