# Supplementary material for: How executives’ expectations and experiences shape population health management strategies
Source: BMC Health Serv Res. 2019 Oct 26;19:757. doi: 10.1186/s12913-019-4513-3 (PMC6815420; doi:10.1186/s12913-019-4513-3)
Supplement: Supplementary file 2 — Interview guideline (DOCX 54 kb) [file 12913_2019_4513_MOESM2_ESM.docx]

**Additional file 2: Interview guideline to identify the short- medium and long expectations of the place-based initiatives, their underlying prior experiences and intended strategies**

Interview questions (60 minutes)

Part 1: sketching a brief picture of the expectations/ambitions outlined at the start of the place-based initiatives.

*1. To what extent do you recognize this description?*

Let us now move on to the short, medium and long term perspectives. First of all, I would like to ask you to write down the short-, medium- and long-term expectations in the relevant timeframe. You have five boxes for each time period in which you can write down an expectation (see format at p.3). You have five minutes to do this. Then we will discuss what you have noted.

5 minutes time to write down the expectations

*2. What is the first thing you have noted under the first period?*

*3. How can these expectations be explained?*

*Specifically (to gain insight into the SCMO relationship):*

*what is this expectation based on?*

*Is this expectation based on previous experiences?*

*If so, what experience? How did this experience arise?*

*What is the underlying assumption?*

*What value or meaning do you think lies beneath this?*

*Given the expectations and prior experiences, what is your intended strategy?*

The researcher then goes through all the aspects noted in the boxes per period in a similar way.

After these aspects have been discussed, the researcher introduces the doc visualisation of the theoretical framework for PHM: CAHN, in order to identify other factors per period.

*4. If you look at this document, are there any other factors that also play a role with regard to the short-term expectations?*

If the interviewee asks a question for clarification, the researcher provides the definition of the component. For the definitions of all components, see p.2.

Repeat questions at 3

The procedure is repeated for the medium and long term expectations.

**CAHN definitions and visualisations of the key components**


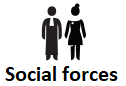
**1.Social Forces**

Social forces anchored at the institutional level consist of three broad types

of forces that supply guidelines for the behaviour of people: cultural-cognitive

(what generally does happen), normative (what should happen) and regulative

(what must happen).


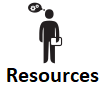
**2.Resources**

The demand and supply side of resources and the technologies available to

organizations, in order for organizations to produce services.


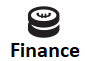
**3.Finance**

The management of financial arrangements, which contains 3 elements: financial

strategies, contractual relationships and contractual scope and requirements.


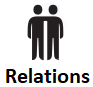
**4.Relations**

How (a new) culture is enacted at the interpersonal level and comprises seven

constructs: trust, mindfulness, heedfulness, respectful interaction, group diversity,

social and task relatedness, and communication effectiveness.


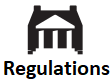
**5.Regulations**

Regulations refers to the national (federal) - state (provincial) and/or county

(municipal) health policy and accompanying laws and regulations and to political

influence, problem streams and the political agenda.


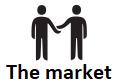
**6.The Market**

The local market refers to 4 elements that influence the working relationships

between organizations within a local health care market (trust-reciprocity-respect;

agreement on purpose and needs; engagement; history of the local market), and

to the structures and dynamics of this local market.


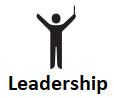


**7.Leadership**

Leadership structures, processes and styles that provide support and direction for

the development of PHM across organizations and sectors.

**
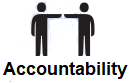
**
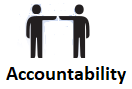
**8.Accountability**

Processes by which one party reports to another on its actions or performance

either with or without consequences, i.e. who, what and how.

**
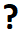
**

**9. Other**

*Identification of the short- middle- and long term expectations*

*Interview……………………………………………………………………………………………...(name). Date …*

| **Short term expectations (until 2018)**  **(5 years after the start of the place-based initiative)** | **Medium term expectations (until 2023)**  **(10 years after the start of the place-based initiative)** | **Long term expectations (until 2033)**  **(20 years after the start of the place-based initiative)** |
| --- | --- | --- |
| **1.** | **1.** | **1.** |
| **2.** | **2.** | **2.** |
| **3.** | **3.** | **3.** |
| **4** | **4.** | **4.** |
| **5.** | **5.** | **5.** |
